# Renal–hepatic–pancreatic dysplasia-1 with a novel *NPHP3* genotype: a case report and review of the literature

**DOI:** 10.1186/s12887-022-03659-7

**Published:** 2022-10-18

**Authors:** Hui Zhu, Zhi-hui Zhao, Shu-yao Zhu, Fu Xiong, Li-hong He, Yong Zhang, Jin Wang

**Affiliations:** 1 Department of Pediatrics, Sichuan Provincial Maternity and Child Health Care Hospital, No. 290 West Second Street, Shayan Road, Chengdu, 610045 Sichuan China; 2 Department of Neonatology, Sichuan Provincial Maternity and Child Health Care Hospital, No. 290 West Second Street, Shayan Road, Chengdu, 610045 Sichuan China; 3 Ultrasonic Department, Sichuan Provincial Maternity and Child Health Care Hospital, No. 290 West Second Street, Shayan Road, Chengdu, 610045 Sichuan China; 4 Department of Medical Genetics and Prenatal Diagnosis, Sichuan Provincial Maternity and Child Health Care Hospital, No. 290 West Second Street, Shayan Road, Chengdu, 610045 Sichuan China

**Keywords:** Renal–hepatic–pancreatic dysplasia, *NPHP3*, Ciliopathy

## Abstract

**Background:**

Renal–hepatic–pancreatic dysplasia type 1 (RHPD1) is a rare sporadic and autosomal recessive disorder with unknown incidence. RHPD1 is caused by biallelic pathogenic variants in *NPHP3*, which encode nephrocystin, an important component of the ciliary protein complex.

**Case presentation:**

In this case report, we describe a male newborn who was confirmed by ultrasound to have renal enlargement with multiple cysts, pancreatic enlargement with cysts, and increased liver echogenicity, leading to the clinical diagnosis of RHPD. In addition, a compound heterozygous pathogenic variant, namely, *NPHP3* c.1761G > A (p. W587*) and the c.69delC (p. Gly24Ala24*11) variant, was detected by WES. The patient was clinically and genetically diagnosed with RHPD1. At 34 h of life, the infant died of respiratory insufficiency.

**Conclusion:**

This is the first published case of RHPD1 in China. This study broadens the known range of RHPD1 due to *NPHP3* pathogenic variants.

## Background

Renal–hepatic–pancreatic dysplasia type 1 (RHPD1) is a rare sporadic and autosomal recessive disorder characterized by hepatic dysgenesis, renal dysplasia and pancreatic fibrosis. RHPD1 is caused by homozygous or compound heterozygous mutations in *NPHP3* that encode nephrocystin [1]. It is sometimes referred to as Ivemark II syndrome (OMIM number 208540). RHPD1 is extremely rare and has an unknown incidence. Here, we report a male newborn with severe *NPHP3*-related ciliopathy with a severe RHPD1 phenotype and a novel genotype that was confirmed by using whole-exome sequencing (WES) and Sanger sequencing verification. Informed consent for genetic testing was obtained from the patient’s parents. This report broadens the known range of RHPD1 due to *NPHP3* pathogenic variants. To summarize the clinical manifestations and genotypes, we also reviewed the RHPD1 cases reported in the literature, from the first reports in 1959 to the most recent.

## Case presentation

**Fig. 1:**
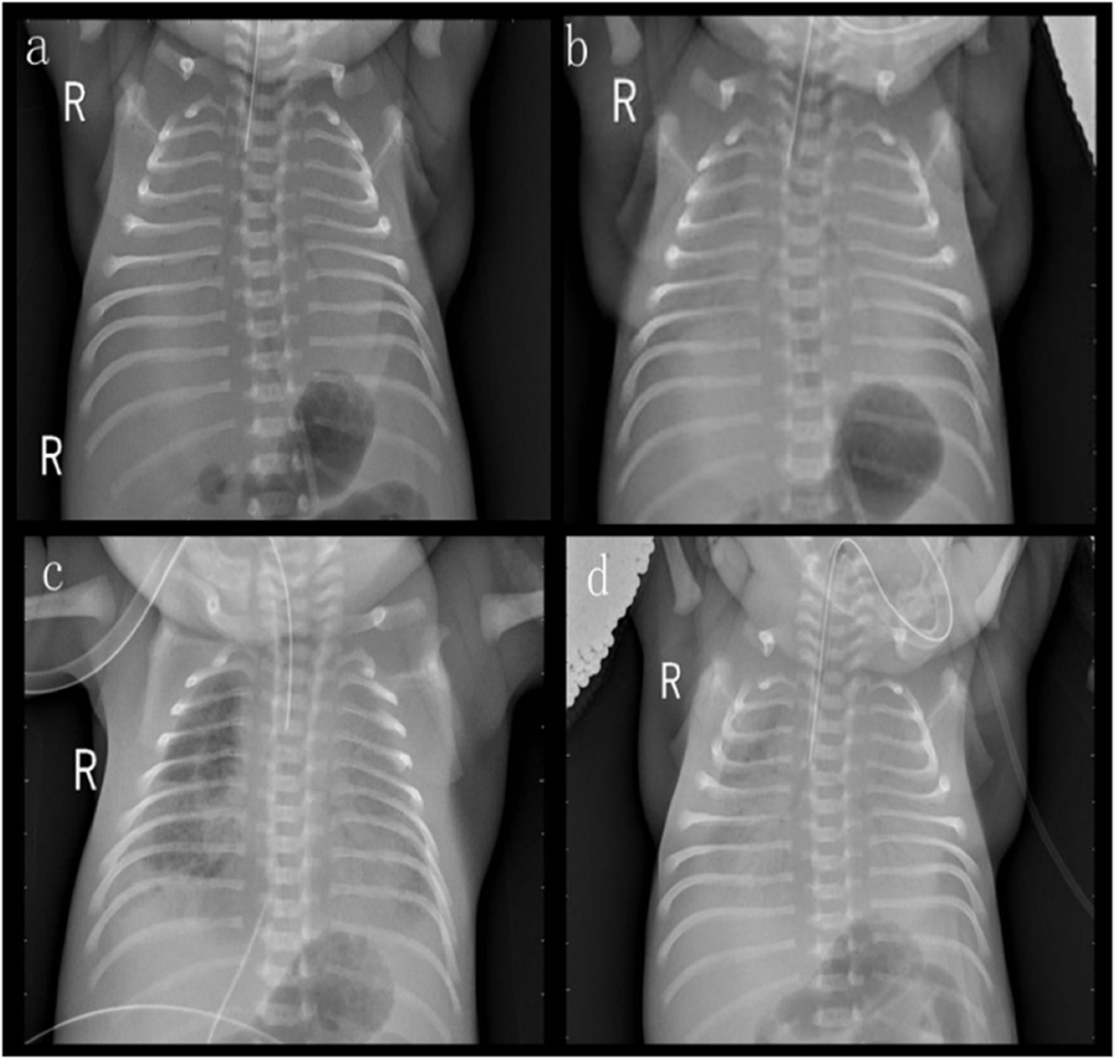
Chest X-ray before treatment documented normal ribs and vertebrae, and the transmittance of both lungs decreased significantly, leading to a presentation of white lung (**a**). After 1 round (**b**), 2 rounds (**c**) and 3 rounds (**d**) of pulmonary surfactant therapy, chest X-ray showed white lung

A male infant was born prematurely at 32^+4^ weeks of gestation by cesarean section due to severe oligohydramnios (amniotic fluid index: 2.8 cm) on prenatal ultrasound. A prenatal ultrasound at 23 weeks of gestation demonstrated multicystic kidneys. An amniocentesis performed for karyotyping revealed normal chromosomes. A tentative diagnosis was made of autosomal recessive polycystic kidney disease. The mother was 22 years of age, and the father was 27 years of age. The clinical examination and abdominal and renal ultrasound of the parents were normal. The couple was nonconsanguineous and of Chinese origin. The parents were from Ya 'an, Sichuan Province, China. There was no history of prenatal exposure to alcohol, drugs, or medications. This was the couple’s second pregnancy. The infant was born normally, and there was no obvious amniotic fluid at delivery. The placenta and umbilical cord were normal. There was no deformity. Apgar scores were 10 at 1 min and 8 at 5 min of age. The birth weight and length were 1680 g and 42 cm, respectively. Physical examination indicated continuous machine-like murmurs and hepatomegaly. The baby developed respiratory distress approximately 10 min after delivery. At 15 min of life, the oxygen saturation acutely decreased from 90 to 50%, and the patient was intubated and mechanically ventilated. In addition, pulmonary surfactant was intratracheally aspirated. A bedside chest radiograph documented normal ribs and vertebrae, and the transmittance of both lungs decreased significantly, leading to a presentation of white lung, in line with neonatal respiratory distress syndrome (NRDS) grade IV (Fig. 1a). The cyanosis and labored breathing of the infant under invasive ventilator-assisted ventilation improved and oxygen saturation gradually increased, but levels could not be maintained in the normal range; the respiratory oxygen concentration was gradually increased to 80%, and oxygen satiety was maintained at approximately 70%. Arterial blood showed severe metabolic acidosis with respiratory acidosis, hypoxemia, and hyperlactacidemia (pH 6.852, PaCO_2_ 47.3 mmHg, PaO_2_ 56.8 mmHg, SO2 74.8%, BE -25.3 mmol/L, lactic acid 11.3 mmol/L). After replacing the oscillating ventilator with auxiliary ventilation, the patient's peripheral oxygen saturation increased to the normal range and was maintained at 93–95%. He was also given sodium bicarbonate to correct the acidosis.

The laboratory results were as follows: serum potassium ion concentration of 6.2 mmol/L, serum-free calcium concentration of 0.62 mmol/L, serum creatinine concentration of 130 μmol/L, BUN level of 6.7 mmol/L, serum β2 microglobulin level of 9.5 mg/L, lactic acid level of 20.5 mmol/L, albumin level of 28.6 g/L, and serum total bilirubin and direct bilirubin levels of 30.3 μmol/L and 12.9 μmol/L, respectively. Repeated monitoring of serum glucose concentration showed fluctuations ranging from 11.2 mmol/L to 17 mmol/L. Routine blood tests and aspartate, alanine aminotransferase, amylase and lipase levels were normal. Routine urine and microscopy examination revealed no protein or red blood cells and no hypercalciuria.

**Fig. 2:**
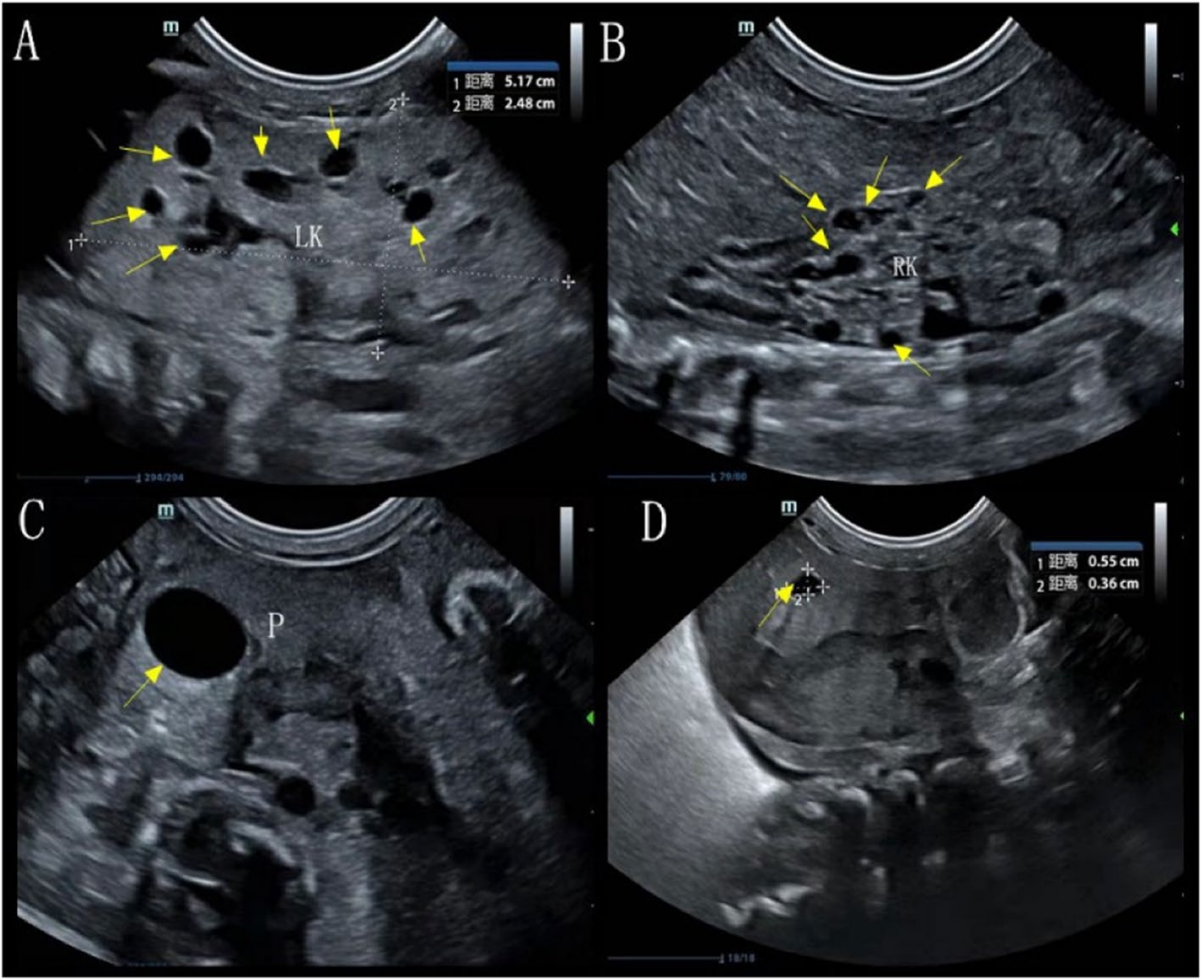
Ultrasound images of the upper abdomen showed the left kidney (arrows) multiple cysts (**A**), the right kidney (arrows) multiple cysts (**B**), (arrows) the head and tail of the pancreas contained two cysts (**C** and **D**)

**Fig. 3:**
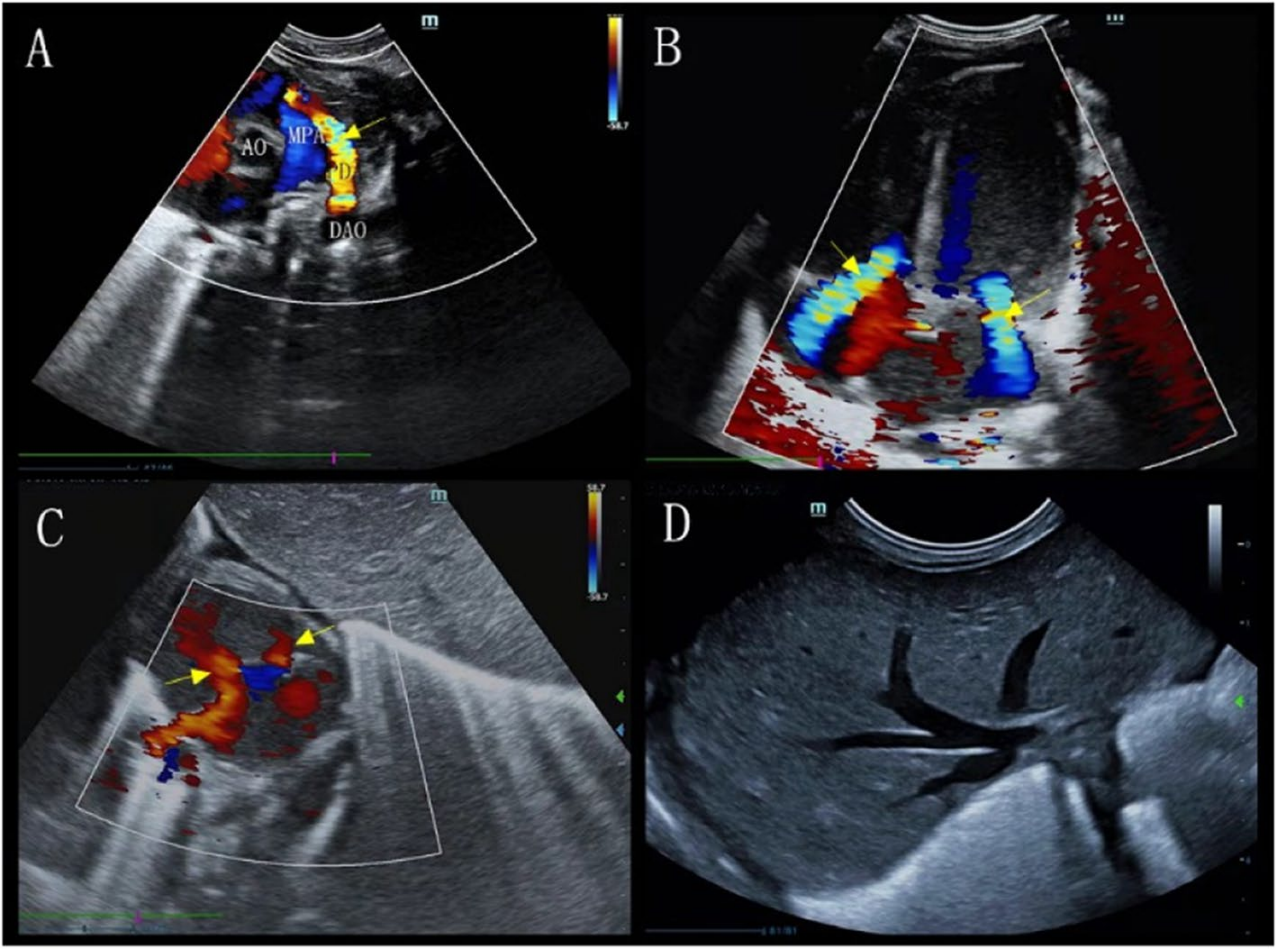
Echocardiography showed (arrow) patent ductus arteriosus (**A**), (arrow) mitral and tricuspid regurgitation (**B**), (arrow) atrial septal defect (**C**), (arrow) patent foramen ovale (**C**). Liver ultrasound showed an uneven liver echogenicity (**D**)

**Fig. 4:**
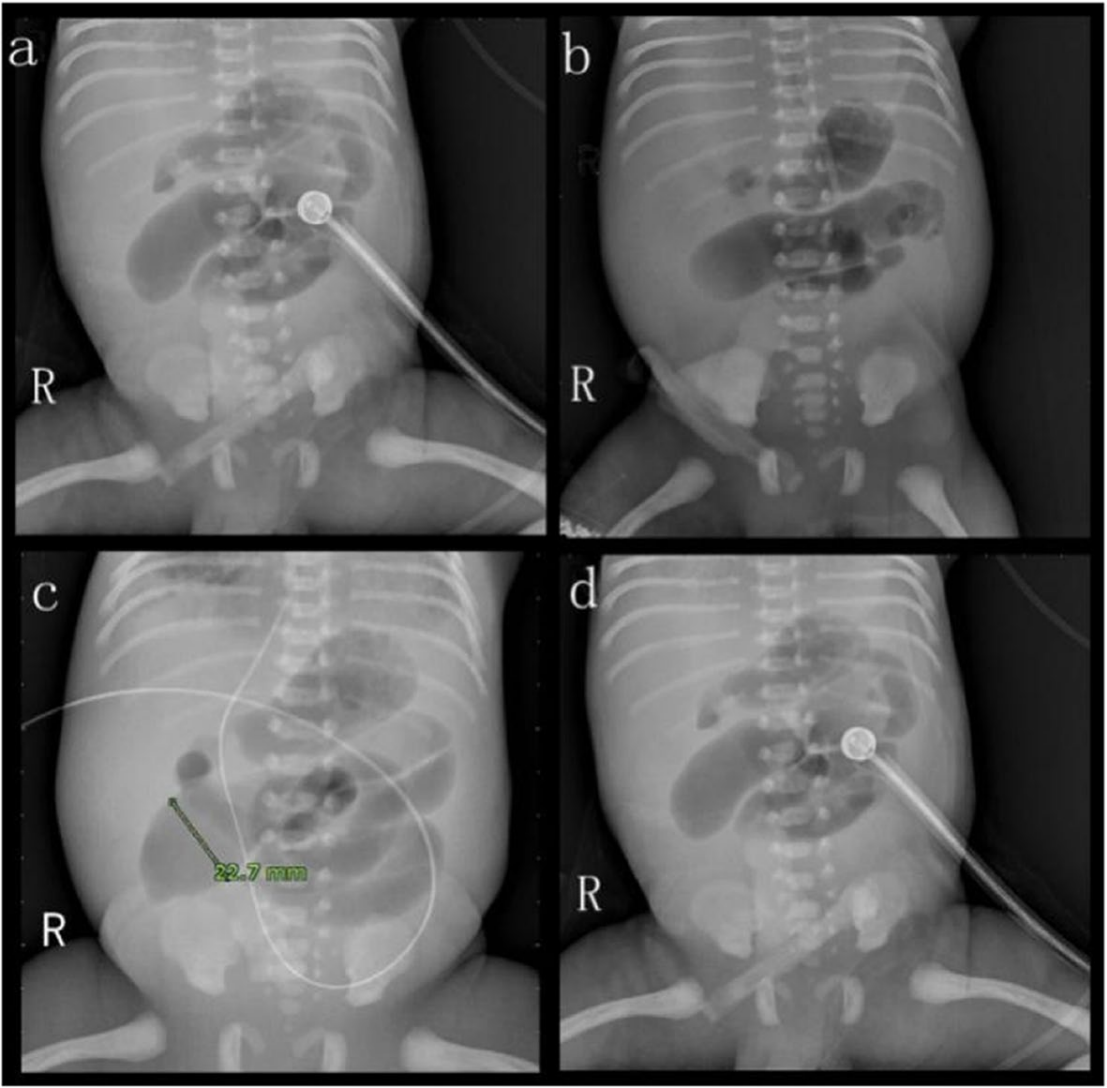
Multiple abdominal X-ray showed that the abdominal bowel was inflated very little and that the colon was not inflated (The examination times of a, b, c and d were consistent with the four chest X-rays)

Renal ultrasound studies showed enlarged kidneys (left kidney: 5.1 cm × 2.8 cm; right kidney: 3.5 cm × 1.5 cm), enhanced echogenicity, and multiple cysts of various sizes (Fig. 2A-B). Ultrasound showed that the bilateral ureters were not dilated and that the bladder was not filled. Ultrasound showed an enlarged pancreas with uneven parenchymal echogenicity enhancement. The head and tail of the pancreas contained two cysts, the larger located in the head measuring 1.2 cm × 1.5 cm (Fig. 2C) and the smaller located in the tail measuring 0.6 cm × 0.4 cm (Fig. 2D). Abdominal ultrasound showed uneven liver echogenicity, no intrahepatic bile duct dilation, and no gallbladder and spleen abnormalities (Fig. 3D). Echocardiography showed pulmonary hypertension, minor pulmonary valve regurgitation, patent ductus arteriosus, and an inner diameter of pulmonary artery end of 3.9 mm. CDFI showed detectable biphasic bidirectional regurgitation, atrial septal defect (2.2 mm) with patent foramen ovale, mild to moderate mitral and tricuspid regurgitation, and reduced left ventricular systolic function (LVEF: 43.5%, LVFS: 19%) (Fig. 3). The cardiac position, myocardial echogenicity, and thickness were normal. The cranial ultrasound was normal. The chest ultrasound showed bilateral pleural effusion (left 1.3 cm, right 0.3 cm). Multiple abdominal X-rays showed little abdominal bowel inflation and an absence of colon inflation (Fig. 4).

The patient experienced repeated severe metabolic acidosis, hyperkalemia, hypocalcemia, and hyperglycemia, which remained difficult to correct after treatment with sodium bicarbonate plus insulin. He had episodic oxygen desaturation and pulmonary hypertension and was treated with nitric oxide, dopamine, dobutamine, and epinephrine. The patient’s repeated hypoxemia and white lung on chest radiographs (Fig. 1b-d) persisted after 3 rounds of pulmonary surfactant therapy.

**Fig. 5:**
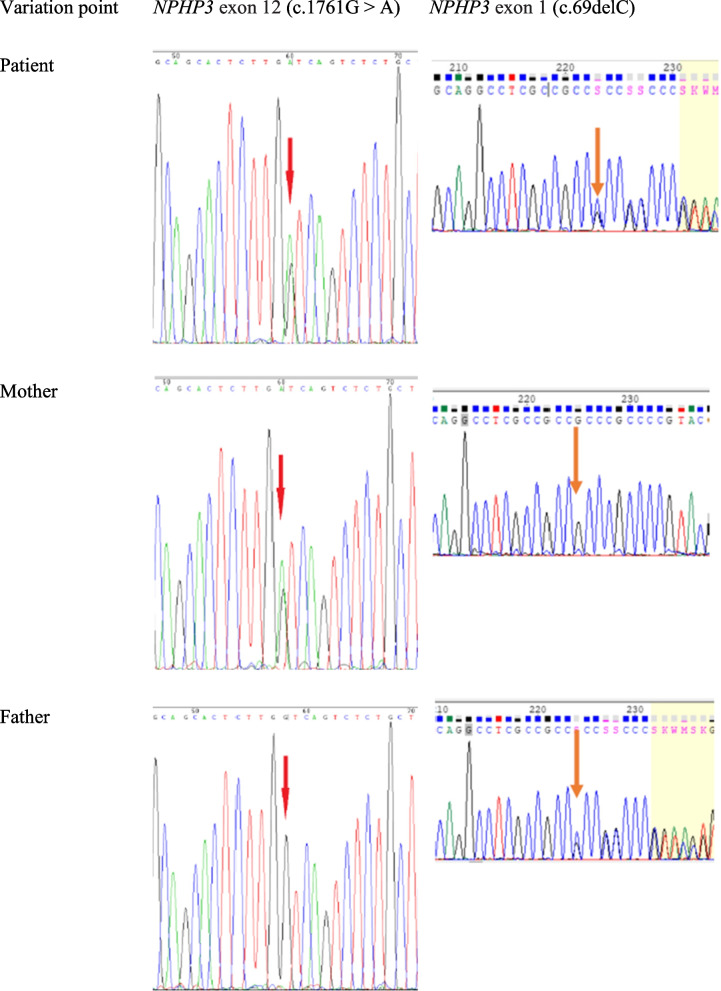
Sanger sequencing showing compound heterozygous mutations in nephrocystin 3 (*NPHP3*): the patient had heterozygous pathogenic variants of both c.1761G > A in exon 12 and c.69delC in exon 1

The infant's urine output was significantly diminished, with a total urine volume of 35 ml from birth to the end of life. The child's condition was critical, and a voiding cystogram was not allowed. Abdominal X-rays indicated little aeration of the abdominal bowel, wherein the colon was not aerated, and there was no fetal fecal discharge at any time. After treatment with kaiserol for defecation, there were a few mucoid substances; thus, it was speculated that the infant had a digestive tract malformation. At 1 day 10 h of life, the infant made a difficult respiratory effort with little chest movement, and his parents elected to withdraw ventilatory support. Possibilities of polycystic kidney and autosomal recessive genetic fatal disease were considered as the differential diagnosis, and blood samples of the child and parents were sent for genetic analysis. We used Roche NimbleGen SeqCap EZ MedExome, which captures approximately 47 Mb human exonic regions, to enrich the whole exome library and perform sequencing on the Illumina NovaSeq 6000 platform. The WES data quality of the proband (21-VEX-107), his mother (21-VEX-108) and his father (21-VEX-109) are shown in Table 1. WES revealed a compound heterozygous pathogenic variant in the *NPHP3* gene on chromosome 3, a nonsense mutation in exon 12 (NM_153240.5: c.1761G > A, p. W587*) and a frameshift mutation in exon 1 (NM_153240.5: c.69delC: p. Gly24Ala24*11). We also confirmed by Sanger sequencing that the nonsense mutation was derived from the father and the frameshift mutation from the mother (Fig. 5). The two variants were not listed in any databases. According to the American College of Medical Genetics (ACMG) guidelines (Genet Med. 2015) [2], *NPHP3* NM_153240.5: c.1761G > A: p. W587* was classified as “Likely Pathogenic” with PVS1 (loss-of-function is the known mechanism of RHPD1 disorder) + PM2 (Absent from controls in genome Aggregation Database, 1000 Genomes Project, or Exome Aggregation Consortium); *NPHP3* NM_153240.5: c.69delC:p. Gly24Ala24*11 was classified as “pathogenic” with PVS1 (loss-of-function is the known mechanism of RHPD1 disorder) + PM2 (absent from controls in the Aggregation Database, 1000 Genomes Project, or Exome Aggregation Consortium) + PM3 (for recessive disorders, detected in trans with a pathogenic variant). We finally diagnosed the patient with RHPD1 both genetically and clinically.

**Table 1:** The WES data quality of proband (21-VEX-107), his mother (21-VEX-108) and father (21-VEX-109)

Sample ID	Raw Bases (Gb)	Aligned Bases(G)	Mean Cov (x)	% Bases > = 20x
21-VEX-107	13.8	12.8	105.6	97.3
21-VEX-108	12.3	11.5	97.5	96.6
21-VEX-109	15.9	14.9	119.0	98.0

## Family history

Delivery of the couple’s first child was induced at 28 weeks after the prenatal ultrasound suggested bilateral renal cysts and severe oligohydramnios. There was a normal male karyotype. Subsequently, labor was induced, and a male fetus was delivered with Apgar scores of 1 at 1 min and 1 at 5 min. Cardiopulmonary resuscitation was not successful. The male fetus has no external dysmorphic features. His family refused further procedures, including autopsy, histopathology and genetic analysis. The couple was unaware of other renal, liver or pancreatic disease cases in their families.

## Discussion and conclusions

RHPD is an extremely rare autosomal recessive disease, with few case reports since the first case description in 1959 by Ivemark et al. [3]. In 2008, Bergmann and his colleagues reported that RHPD is caused by biallelic pathogenic variants in *NPHP3* that encode nephrocystin [1]. Homozygous or compound heterozygous pathogenic variants of *NPHP3* on chromosome 3q22 lead to RHPD1 (OMIM number 208540).

RHPD1 is characterized by hepatic dysgenesis, renal dysplasia and pancreatic fibrosis. Additionally, liver biopsy showed ductal malformation characteristic of RHPD1 [4]. Subsequently, all cases of RHPD with the above renal, hepatic and pancreatic characteristics confirmed by autopsy and histopathological examination without genetic analysis were considered RHPD1 [5–7]. RHPD was first described in 1959 by Ivemark et al., and only 52 cases have been reported since, including the present case. Only 14 of the 52 patients underwent gene analysis (►Table 2). It should be added that in Table 2, patient 3 and patient 4 reported by Bergmann C et al. [1] in 2008 were the same cases reported by Neuhaus T J et al. [8] in 1996. No genetic analyses were performed on these RHPD patients—they were diagnosed by histology and autopsy [9–16]. Among the 38 cases, Larson R S et al. [16] reviewed 21 similar cases and reported 2 families with a total of 3 new cases.

**Table 2:** Clinical manifestation and pathogenic variants of RHPD1 have been previously reported

**Reference**	**Pathogenic variants**	**Renal**	**Hepatic**	**Pancreatic**		
[5]	Homozygous: c.2975C > T(p.Ala992Val)	High echogenicity	Enlarged	Amylase and lipase levels were elevated but normal on MRCP; no biopsies		
[6]	Heterozygous: c.1817G > A (p.Trp606Ter); c.3402_3403delTG (p.Ala1135SerfsTer5)	High echogenicity and absent corticomedullary differentiation	Enlarged with dysplasia of the bile ducts	Normal on ultrasound and no biopsies		
[17]	Homozygous: c.1985 + 5G > A	Cysts	Enlarged, fibrosis	Normal, no autopsy		
[7] Patient 1	Heterozygous: c.1206delA (p. Val403Serfs*9); c.3003delT (Phe1001Leufs *61)	Cystic dysplastic	Bile duct paucity, choledochal cyst	Fibrosis, dilated irregular ducts		
[7] Patient 2	Heterozygous: c.1206delA (p. Val403Serfs*9); c.3003delT (Phe1001Leufs *61)	Cysts	Congestion	Fibrosis, dilated irregular ducts		
[7] patient 3	Heterozygous: c.1206delA (p. Val403Serfs*9); c.3003delT (Phe1001Leufs *61)	Enlarged, cystic dysplastic	Bile duct paucity, portal, fibrosis, hepatic lobe cyst, enlarged common bile duct	Fibrosis, dilated irregular ducts		
[4] Patient 1	Homozygous: c.2694-2_2694-1delAG	Small, cystic dysplastic	Liver fibrosis and bile duct paucity	Enlarged, fibrosis		
[4] Patient 2	Homozygous: c.2694-2_2694-1delAG	Enlarged, cystic dysplastic	Bile duct paucity, abnormally branched, medium-size bile ducts	Dysplasia, dysplastic ducts		
[1] Patient 1	Homozygous: c.1729C > T (p. Arg577X)	Enlarged, multicystic dysplastic	Cysts	Unknown		
[1] Patient 2	Homozygous: c.1729C > T (p. Arg577X)	Enlarged, multicystic dysplastic	Ductal plate malformation and congenital hepatic fibrosis	Cysts and fibrosis		
[1] Patient 3	Heterozygous: c.2918G to A (p.Arg973Gln); c.3340C > T (p.Gln1114X)	Multicystic dysplastic	Ductal plate malformation and congenital hepatic fibrosis	Normal		
[1] Patient 4	Heterozygous: c.2918G > A (p.Arg973Gln); c.3340C > T (p.Gln1114X)	Enlarged, multicystic dysplastic, vesico-ureteric reflux into grossly	Enlarged	Increased echogenicity		
[1] Patient 5	Homozygous: c.1985 + 5G > A	Glomerulocystic kidney disease	Hepatopathy with cholestasis, cirrhosis and portal hypertension	Pancreatic amylase constantly increased, normal endocrine and exocrine function		
**Reference**	**CNS**	**Cardiac**	**Chest**	**Situs inversus**	**Skeletal**	**Other**
[5]	Normal	Normal	Normal	No	Normal	Splenomegaly
[6]	Normal	peripheral pulmonary artery stenosis	Normal	No	Normal	Normal
[17]	Normal		Normal	No	Normal	hypothyroidism
[7] patient 1	Choroid plexus cyst	Normal	Pulmonary hypoplasia and a bell shaped rib cage	No	Hypocalvaria, large fontanelles, wide cranial sutures, widened growth plates, abnormal development of the trabeculae of the ribs, handle-bar clavicles, wedge defects of the thoracic vertebrae	Persistent transfusion-dependent anemia
[7] patient 2	Anencephaly	Normal	Normal	No	Unknown	Normal
[7] patient 3	Poorly developed brain with bilateral exposure of the insulae	PFO, PDA RVH	Normal	No	Hypocalvaria, large fontanelles	
[4] Patient 1	Normal	Normal	Lung hypoplasia	No	A short sternum	Potter facies
[4] Patient 2	Triangular configuration of lateral ventricles, polymicrogyria of the cingulated gyri	Dextroposition	Lung mirror	Yes	bilateral symmetrical flexion contractures of multiple joints	Potter facies
[1] Patient 1	Cyst in right ventricle, Bilateral choroid plexus cyst	ASD, PDA, RVH	Unknown ext	No	Normal	Unknown
[1] Patient 2	Normal	PDA, nodular dysplasia of valves	Unknown	No	Hypocalvaria, large fontanelles	Unknown
[1] Patient 3	Normal	Normal	Lung hypoplasia and hyaline membrane disease	No	Normal	Normal
[1] Patient 4	Normal	Normal	Normal	No	Normal	Splenomegaly
[1] Patient 5	Normal	Normal	Normal	Yes	Postaxial polydactyly left foot	Bilateral preauricular fistulas

*NPHP3* is part of the ciliary protein complex required for proper renal and cardiovascular development [18]. Ciliopathies are a group of disorders that result from ciliary dysfunction. *NPHP3* pathogenic variants can cause multisystemic diseases that affect multiple organs, including the kidneys, livers, pancreas, central nervous system, structural heart, situs inversus and skeleton [19]. The clinical and genetic findings that can be seen in RHPD1 are listed in Table 2. Among the cases reported thus far, including the present case, there are only two children with the same genotype who have shown similar phenotypes; these two cases were from different families with different nationalities and were reported in different calendar years. Among the previously reported cases, abnormal pancreatic clinical manifestations of RHPD1 are limited to two children with insulin-dependent diabetes mellitus [15, 20]. Our patient also had insulin-dependent diabetes. In this case, prenatal ultrasound suggested oligohydramnios, and there was almost no amniotic fluid at birth. After several rounds of pulmonary surfactant treatment, repeated hypoxemia persisted, and chest X-rays suggested white lung (Fig. 1). Therefore, the patient was presumed to have pulmonary dysplasia. Moreover, the patient survived for 34 h without spontaneous defecation, and no defecation was observed after anal inclusion with kaiserol. X-ray of the abdomen indicated that the intestine was less inflated and that the colon was not inflated (Fig. 4); thus, digestive tract malformation (such as intestinal atresia) was possible.

*NPHP3* pathogenic variants can result in RHPD1, isolated nephronophthisis, Senior-Loken syndrome, Meckel-Gruber-like syndrome (MKS), and embryonic lethality [1]. Previous reported cases have shown that there may be considerable phenotypic overlap resulting from pathogenic variants of the different *NPHP* genes and genes associated with the other ciliopathies. Comparable or identical mutations in the same gene cause very different phenotypes [7]. RHPD is now known to be a component of many other well-defined syndromes, such as Zellweger, Meckel, Jeune, Elejalde, and Saldino–Noonan chondrodysplasias, trisomies 9 and 13, and glutaric aciduria II [7]. Although the renal and hepatic pathologies in RHPD are identical to those observed in some other ciliopathies (e.g., MKS, isolated nephronophthisis, Senior–Loken syndrome, Goldston syndrome, Joubert syndrome), RHPD differs in terms of its pancreatic involvement and the absence of more specific anomalies of the other ciliopathies. Among these, the minimal diagnostic criteria of MKS are most often formulated as the presence of at least two of the three main manifestations, i.e., bilateral renal cystic dysplasia, occipital encephalocele or other anomalies of the central nervous system, and polydactyly [21]. Histologically or microscopically, the phenotypes of the kidney, liver and pancreas in the previously described clinical syndromes only partially overlap with RHPD, with no consistent manifestations. Therefore, RHPD1 can be differentiated clinically, histologically and molecularly from other ciliated diseases [1].

It is also noteworthy that Jordan P et al. [22] recently reported a case of RHPD caused by a c.600-2A > C homozygous variation in *DNAJB11 (*NM_016306.5), affecting the consensus acceptor splice site of intron 5. Nonetheless, RHPD associated with the *DNAJB11* diallelic loss-of-function mutation is not in the OMIM database to date. Kapur R P et al. reported a case of RHPD associated with placental mesenchymal dysplasia (PMD) and androgenetic-biparental mosaicism (ABM) [23]. Mutational analysis of coding sequences did not reveal any pathogenic variant in *NPHP3*; nonetheless, the fetal phenotype and laser capture data support the model of a paternally transmitted autosomal recessive disorder that occurred because of ABM. Frank et al. reported a case of RHPD2 caused by a *NEK8* pathogenic variant; however, this case had a phenotype consistent with RHPD1 [24, 25]. Pathogenic variants in *NEK8* on chromosome 17q11 result in RHPD2. At present, *NEK8* deficiency should be considered a cause of bile duct paucity with cystic renal disease, with or without congenital heart malformations [26]. In summary, the above three cases were pathologically consistent with RHPD, but no *NPHP3*-related gene pathogenic variants were found. Thus, RHPD may be a component of the phenotype of some diseases. Therefore, these diseases can only be identified by molecular characterization for RHPD cases with the same phenotype [26].

A compound heterozygous pathogenic variant in *NPHP3* was detected in our patient (Fig. 5). This variant was not listed in any databases. In addition, no candidate genes other than *NPHP3* were found in this patient by WES. Although the autopsy and pathological tissue analysis of our case were not completed due to the family's refusal, we finally diagnosed the patient with RHPD1 both genetically and clinically. Neither autopsy nor genetic analysis were performed on the deceased elder brother, who likely had the same disease.

The majority of patients with RHPD1 expire in the perinatal period or during the early neonatal period as a result of respiratory insufficiency secondary to pulmonary hypoplasia [17]. Only a few patients have survived due to combined liver–kidney transplantation in early childhood, and these infants had no oligohydramnios in utero and no pulmonary hypoplasia after birth [1, 8]. Our patient also died of respiratory insufficiency and could not be saved with ventilators and pulmonary surfactants [1, 12]. Due to the failure to form significant amounts of fetal urine, the amount of amniotic fluid surrounding the fetus was too low, a condition known as oligohydramnios. Severe and prolonged oligohydramnios leads to a constellation of findings that include pulmonary hypoplasia, Potter's facies, and positional abnormalities [1, 12].

Because the recurrence risk for a couple with a baby with RHPD1 is 25%, a DNA-based prenatal diagnostic test that can be applied early in subsequent pregnancies is often desired. Molecular diagnosis will have important consequences for genetic counseling and possible prenatal testing. WES is useful to comprehensively analyze known causative genes for kidney disease, including ciliopathies, to identify novel disease genes and to be able to offer genetic counseling as precisely as possible [27].

In conclusion, this is the first published case of RHPD1 in China. This case broadens the known range of RHPD1 due to *NPHP3* pathogenic variants. Although the diagnostic value of autopsy is high, it is difficult to obtain informed consent from family members or guardians in our country. Therefore, comprehensive auxiliary examination and genetic examination are also extremely important.

## Data Availability

All the data and materials used are included in the manuscript. The datasets used and/or analyzed during the current study are available from the corresponding author on reasonable request. Our pathogenic variant data has been deposited into the ClinVar bank: https://www.ncbi.nlm.nih.gov/clinvar/ or https://www.ncbi.nlm.nih.gov/clinvar/submitters/508739. And the relevant accession numbers: SCV002571101 and SCV002571102.

## Abbreviations

RHPD1: Renal–hepatic–pancreatic dysplasia type 1
WES: Whole-exome sequencing
PMD: Placental mesenchymal dysplasia
ABM: Androgenetic-biparental mosaicism
